# Sulfated Bile Acids in Serum as Potential Biomarkers of Disease Severity and Mortality in COVID-19

**DOI:** 10.3390/cells13181576

**Published:** 2024-09-19

**Authors:** Emanuele Porru, Rossana Comito, Nicolò Interino, Andrea Cerrato, Marco Contoli, Paola Rizzo, Matteo Conti, Gianluca Campo, Savino Spadaro, Cristiana Caliceti, Federico Marini, Anna L. Capriotti, Aldo Laganà, Aldo Roda

**Affiliations:** 1 Occupational Medicine Unit, Department of Medical and Surgical Sciences, Alma Mater Studiorum, University of Bologna, 40138 Bologna, Italy; emanuele.porru2@unibo.it; 2 Division of Occupational Medicine, “IRCCS” Azienda Ospedaliero—Universitaria di Bologna, 40138 Bologna, Italy; rossana.comito@aosp.bo.it; 3 Laboratorio di Proteomica Metabolomica e Chimica Bioanalitica, “IRCCS” Istituto delle Scienze Neurologiche di Bologna, 40139 Bologna, Italy; nicolo.interino@ausl.bologna.it; 4 Department of Chemistry, “Sapienza” University of Rome, 00185 Rome, Italy; andrea.cerrato@uniroma1.it (A.C.); federico.marini@uniroma1.it (F.M.); annalaura.capriotti@uniroma1.it (A.L.C.); aldo.lagana@uniroma1.it (A.L.); 5 Respiratory Section, Department of Morphology, Surgery, and Experimental Medicine, University of Ferrara, 44121 Ferrara, Italy; marco.contoli@unife.it; 6 Laboratory for Technologies of Advanced Therapies “LTTA”, Department of Translaqutional Medicine, University of Ferrara, 44121 Ferrara, Italy; paola.rizzo@unife.it; 7 Maria Cecilia Hospital, GVM Care & Research, 48022 Cotignola, Italy; 8 Local Unit of Imola, Department of Public Health, Health Service of the Emilia-Romagna Region, 40026 Imola, Italy; matteo.conti@ausl.imola.bo.it; 9 Cardiovascular Institute, Azienda Ospedaliero, University of Ferrara, 44124 Ferrara, Italy; gianlucacampo@unife.it; 10 Intensive Care Unit, Department of Morphology, Surgery, and Experimental Medicine, University of Ferrara, 44121 Ferrara, Italy; savino.spadaro@unife.it; 11 Department of Biomedical and Neuromotor Sciences, Alma Mater Studiorum, University of Bologna, 40123 Bologna, Italy; cristiana.caliceti@unibo.it; 12 Biostructures and Biosystems National Institute “INBB”, 00136 Rome, Italy; 13 Interdepartmental Centre for Industrial Agrofood Research-CIRI Agrofood, University of Bologna, 47521 Cesena, Italy; 14 Interdepartmental Center of Industrial Research “CIRI”-Energy and Environment, Alma Mater Studiorum, University of Bologna, 40126 Bologna, Italy; 15 Department of Chemistry “G. Ciamician”, Alma Mater Studiorum, University of Bologna, 40126 Bologna, Italy

**Keywords:** targeted metabolomics, untargeted metabolomics, COVID-19, early severity biomarker, bile acids

## Abstract

The fight against coronavirus disease 2019 (COVID-19) continues. Since the pandemic’s onset, several biomarkers have been proposed to assess the diagnosis and prognosis of this disease. This research aimed to identify potential disease severity biomarkers in serum samples of patients with COVID-19 during the disease course. Data were collected using untargeted and targeted mass spectrometry methods. The results were interpreted by performing univariate and multivariate analyses. Important metabolite classes were identified by qualitative untargeted metabolomics in 15 serum samples from survivors of COVID-19. Quantitative targeted metabolomics on a larger patient cohort including 15 non-survivors confirmed serum 3-sulfate bile acids (i.e. GLCA-3S) were significantly increased in non-survivors compared to survivors during the early disease stage (*p*-value < 0.0001). Notably, it was associated with a higher risk of mortality (odds ratio of 26). A principal component analysis showed the ability to discriminate between survivors and non-survivors using the BA concentrations. Furthermore, increased BA-S is highly correlated with known parameters altered in severe clinical conditions.

## 1. Introduction

Coronavirus disease 19 (COVID-19) is an infectious disease caused by the SARS-CoV-2 virus that has spread rapidly worldwide since 2019, challenging the resilience of healthcare systems in most countries. Severe COVID-19 is usually characterized by respiratory compromise and multiorgan failure [1]. Several clinical biomarkers change during the infection, with a correlation between disease severity and survival probability. Although the markers are considered non-specific, acute-phase reactants, including C-reactive protein (CRP), ferritin, serum amyloid A, and procalcitonin (pct), have been reported as sensitive markers of acute COVID-19 disease [2]. Many studies have identified the association between severe COVID-19 and heightened platelet activation [3] as well as deficient interferon (IFN)-α levels have been also associated with increased interleukin (IL)-10 expression in patients progressing to severe or life-threatening COVID-19 conditions [4]. Significantly elevated white blood cell count, marked lymphopenia, high neutrophil count, thrombocytopenia, and significantly elevated inflammatory biomarkers, such as γ’ fibrinogen, were associated with severe disease and the risk of developing sepsis with rapid progression [2,5]. Endocan also showed encouraging prospects as a prognostic laboratory test [6]. In terms of predictive biomarkers, Park et al. reported that the soluble suppression of tumorigenicity-2 could be a useful biomarker to predict ICU (intensive care unit) admission, ventilator use, and ECMO (extracorporeal membrane oxygenation) use [7]; early transforming growth factor (TGF)-β serum levels in patients with COVID-19 predict disease severity and fatality [8], while pct appears to be a reliable biomarker for predicting outcomes and adjusting therapies appropriately [9]. One of the main COVID-19 symptoms is acute respiratory distress syndrome (ARDS). In this case, serum levels of angiopoietin-2 and intercellular adhesion molecule (ICAM)-1 were found to be statistically higher in non-survivors than survivors, highlighting their potential role as mortality predictors [10]. In addition, vascular adhesion molecule (sVCAM)-1 was independently associated with mortality [11].

Bile acids (BAs) have been recognized as endocrine molecules that, in addition to facilitating the absorption of fat-soluble nutrients, regulate numerous metabolic processes, including glucose, lipid, and energy homeostasis, as well as inflammation responses [12]. The analysis of BA composition in the biological matrix is improved with new and more performant technologies with higher detectability and specificity. New and unexplored BA molecules have been recently discovered and reported in the literature, such as Oxo-BA [13] in stools and intestinal content and complete sulfated BA (BA-S) patterns [14]. The concentration and composition of BA and BA-S are altered in pathological conditions, especially with liver, intestine, and kidney failure [15,16,17]. BAs, due to their cytotoxicity, often play a role in promoting cancer angiogenesis. They stimulate the secretion of pro-angiogenic factors and recruit angiogenic progenitor cells[18]. Recently, lipidomic profiles of COVID-19-positive, COVID-19-negative patients hospitalized with other infectious or inflammatory diseases, and healthy volunteers were studied. Secondary BAs were found to be one of the parameters with the greatest ability to discriminate between COVID-19-positive and COVID-19-negative patients [19]. In another study using serum samples from healthy and SARS-CoV-2-positive male and female patients, glycocholate sulfate was not found to be altered in females but strongly elevated in males [20]. Other studies reported a higher total BA serum level in patients with COVID-19 than in non-positive individuals [21,22].

BAs have been identified as key modulators of ACE2 expression, the receptor through which SARS-CoV-2 enters human cells, thereby influencing the course of COVID-19 infection. In addition, BAs play a role in various mechanisms, including immunomodulatory effects, inflammation, and metabolism, which contribute to the regulation of the disease. They also affect the function of immune cells, such as macrophages and T cells, acting as modulators of inflammatory responses [22].

The overall aim of the current study was to identify early disease severity biomarkers in a group of patients with COVID-19.

Firstly, we carried out an untargeted high-resolution metabolomics screening of serum samples from COVID-19 survivors at different stages of the disease. According to the results of the untargeted analysis, a subsequent targeted metabolomics analysis for the qualitative and quantitative characterization of a broad spectrum of molecules, specifically BA and BA-S was performed. The target metabolomic study involved a larger cohort of COVID-19 patients, including non-survivor subjects. We looked for the correlation between different clinical parameters and disease severity. This work was developed during the first spread of the SARS-CoV-2 virus; consequently, the subjects in the study were all unvaccinated.

## 2. Materials and Methods

### 2.1. Chemicals

#### 2.1.1. Untargeted Metabolomics

Creatinine, taurine, putrescine, dopamine, guanosine, cystine, benzoic acid, formestane, dihydrotestosterone (DHT) glucuronide, and taurocholic acid were purchased from Sigma Aldrich (Milan, Italy). Isotopically labeled caffeine, creatinine, phenylalanine, benzoic acid, estradiol, estradiol sulfate, and estradiol glucuronide were purchased from Sigma Aldrich (Milan, Italy). Ultrapure grade water and formic acid were purchased from Fischer Scientific (Waltham, MA, USA); ultrapure grade methanol (MeOH) was from Romil Pure Chemistry (Cambridge, UK). LC pure grade ethanol (EtOH) was purchased from Merck (Burlington, MA, USA). The external and internal standard stock solutions were prepared in MeOH at 1 mg mL-1 and stored at −20 °C until use. The external standard work solution, containing creatinine, taurine, putrescine, dopamine, guanosine, cystine, benzoic acid, formestane, dihydrotestosterone (DHT) glucuronide, and taurocholic acid, was prepared at 0.1–50 µg mL^−1^ in H_2_O/MeOH 80:20 (*v/v*) by dilution with ultrapure water and stored at −20 °C. The internal standard work solution, containing isotopically labeled caffeine, creatinine, phenylalanine, benzoic acid, estradiol, estradiol sulfate, and estradiol glucuronide, was prepared at the final concentrations of 0.1–5 µg mL^−1^ H_2_O/MeOH 80:20 (*v/v*) by appropriate dilution and stored at −20 °C until use.

#### 2.1.2. Targeted Metabolomics

Pure chemical standards of cholic acid (CA), chenodeoxycholic acid (CDCA), deoxycholic acid (DCA), ursodeoxycholic acid (UDCA), lithocholic acid (LCA), their respective glycine and taurine conjugates, glycolithocholic acid 3-sulfate, lithocholic acid 3-sulfate, glycochenodeoxy acid 3-sulfate, and isotopically labeled internal standards (Deuterated Bile Acids mixture, SMB00967) were purchased from Sigma-Aldrich (Saint Louis, Missouri, MO, USA). Methanol (CH_3_OH) and acetonitrile (ACN) of HPLC grade (Lichrosolv^®^) were purchased from Merck (Darm-stadt, Germany). Ammonium hydroxide (98% pure) was purchased from Fluka (Buchs, Switzerland). Water of HPLC-MS grade (Millipore) was produced using the depurative system Milli-Q Synthesis A 10 (Molsheim, France). C18 SPE columns (500 mg, 6 mL) were purchased from SiliCycle (Quebec, QC, Canada). Other solvents were all of analytical grade.

Stock solutions of each analyte and internal standard (IS) were prepared in methanol at a concentration of 1 mg/mL and stored at −20 °C. These stock solutions were further diluted in methanol to obtain working solutions containing all the analytes used for calibration curves. Human serum without BA was used to quantify serum samples with clean-up following the solid-phase extraction (SPE) procedure (see Sample Preparation, Section 2.2.2).

### 2.2. Sample Preparation

#### 2.2.1. Untargeted Metabolomics

Before the analysis, serum samples were thawed at room temperature. Subsequently, EtOH was added to 100 µL of serum (final ratio EtOH/H_2_O 4:1, *v/v*) for simultaneous protein precipitation and virus inactivation and then centrifuged for 20 min at 14,000× *g* to remove the protein pellets. The EtOH was removed by evaporation using a Speed-Vac SC 250 Express (Thermo 164 Avant, Holbrook, NY, USA). The quality control (QC) samples were obtained by pooling 20 µL of each sample included in the study. For each sample (serum samples and QCs), 15 µL of ultrapure water and 5 µL of internal standard work solution were added (final solvent mixture: H_2_O/MeOH 99:1, *v/v*). The blank samples consisted of H_2_O/MeOH 99:1 (*v/v*). UHPLC–HRMS analyses were performed after randomization of the samples.

#### 2.2.2. Targeted Metabolomics

Pooled drug-free human serum was purified using activated charcoal to remove endogenous BA as previously reported in the literature [23] and described in the Supplementary Materials section. Sample preparation was performed as previously reported with minor modifications [24]. The 100 µL aliquots of serum samples or human BA-free serum were fortified with 10 µL of the internal standard working solution and were diluted by adding 2 mL of NaOH 0.1N and heated to 64 °C for 30 min, then extracted with SPE.

### 2.3. Untargeted Metabolomics Workflow

A Vanquish binary pump H (Thermo Fisher Scientific, Bremen, Germany) was coupled to a hybrid quadrupole-Orbitrap mass spectrometer Q Executive (Thermo Fisher Scientific) via a heated ESI source. Samples and QCs were analyzed both in positive and negative ion mode. The raw data obtained from the analysis of samples, QCs, and blanks were pre-processed using the software Compound Discoverer version 3·1 (Thermo Fisher Scientific). Biomarker identification based on untargeted metabolomics was performed using a chemometric classification strategy based on coupling multi-level partial least squares discriminant analysis (ML–PLSDA), feature reduction by means of the covariance selection (CovSel) algorithm, and repeated double cross-validation (rDCV). The untargeted workflow, including LC–MS conditions, data pre-processing, statistical analysis, and compound annotation, are fully reported in the Supplementary Materials section.

### 2.4. Targeted Metabolomics Workflow

Liquid chromatography was performed using a 2690 Alliance system (Waters, Milford, MA, USA). Analyses were performed with a triple quadruple mass spectrometer (Quattro-LC, Micromass, Manchester, UK) operating in the multiple reaction monitoring (MRM) acquisition mode. The data were managed and processed using MassLinx V4·0 software (Waters, Milford, MA, USA). Univariate analysis and producing the correlation matrix were performed using GraphPad Prism 8·0·2 software. The variables normally distributed were compared using the t-test and 1-way ANOVA; otherwise, the Mann–Whitney and Kruskal–Wallis tests were used; the significance level was 95%. Multiple variable analyses were performed by analyzing the constructed correlation matrix using Spearman's coefficient with BA and all parameters examined on serum samples. A receiver operating characteristic (ROC) curve was created to evaluate biomarker sensitivities and specificities in predicting clinical conditions. Chemometric analysis was performed with the R-based software CAT (Chemometric Agile Tool release 8 September 2024, Gruppo di Chemiometria Italiana, Italy). The targeted workflow, including LC–MS conditions, data pre-processing, statistical analysis, and compound annotation, are fully reported in the Supplementary Materials section.

### 2.5. Study Design and Study Population

The serum samples investigated in this study were from patients enrolled in the “Pro-thrombotic status in patients with SARS-CoV-2 infection” study (ATTACCo, https://www.clinicaltrials.gov/, accessed on 22 December 2022, identifier NCT04343053). It was a single-center, prospective study, including 54 patients admitted to the hospital for moderate–severe respiratory failure and were positive for SARS-CoV-2 infection. The study aimed to investigate markers of coagulation, the immune response, and endothelium damage at three time points following patients’ hospitalization. The serum samples were obtained at three different times, at inclusion (T1), after 7 ± 2 days (T2), and 14 ± 2 days (T3) [10]. The study population characteristics and clinical and biochemistry markers investigated are described in the studies previously published [4,10,11,25].

Inclusion criteria were as follows: (a) age > 18 years and (b) confirmed SARS-CoV-2 infection (using PCR-positive nasopharyngeal swab specimens). Patients were recruited from March 1 to the end of April 2020.

Clinical management was in accordance with guidelines and specific recommendations for the COVID-19 pandemic by health authorities and scientific societies.

In this study, we conducted a high-resolution untargeted metabolomics study on serum from fifteen survivor patients. The sample amount of the excluded subjects was insufficient for the mass spectrometry analysis. We had eleven samples at time T1 and T2 and ten at time T3. The untargeted metabolomics analysis focused mainly on compounds with a molecular weight between 100 and 800 Daltons, considering all possible multi-charges. Based on the results obtained, we performed a targeted metabolomics study of BA and metabolites in a larger cohort of patients. Serum samples from fifteen survivors were analyzed with a further fifteen non-survivors at T1, nine non-survivors at T2, and seven non-survivors at T3. A multivariate analysis was used to evaluate the relationship between various clinical and biochemical indicators of heart, lung, kidney, and liver damage or immune deficiency and BA metabolites.

## 3. Results

### 3.1. Study Population

Fifteen survivor patients with COVID-19 were included for the untargeted and targeted metabolomics. Patients were mainly male (12/15, 80%) and the most common comorbidities were hypertension (7/15, 47%), a value ≥ 2 for the CHA₂DS₂-VASc score for atrial fibrillation stroke risk (11/15, 73%), peripheral artery disease (5/15, 53%), chronic kidney disease (8/15, 53%), and cancer (4/15, 22%). Non-survivors were older than survivors (71 ± 8 vs 61 ± 9 years, *p*-value 0.04), whereas no significant difference was found in the comorbidities group and the home medical therapy group (*p* > 0.05, Table 1).

Other clinical and biochemical parameters at inclusion are reported in Table S3 in the Supplementary Materials section. At inclusion, there were significative differences between survivors and non-survivors in blood oxygen level (spO2 ahb) (*p*-value 0.02), lymphocytes (*p*-value 0.02), monocytes (*p*-value 0.03), hematocrit (*p*-value 0.04), hemoglobin (*p*-value 0.05), creatinine clearance (*p*-value 0.03), procalcitonin (*p*-value 0.01), tissue factor (*p*-value 0.04), PAI-1 (*p*-value 0.01), lactic acid (*p*-value 0.002), IL-10 (*p*-value 0.0002), and sVCAM-1 (*p*-value 0.002). All patients enrolled in the study were admitted to the hospital for moderate–severe respiratory failure with respiratory parameters at inclusion reported in the Supplementary Materials section (Table S3). Some of the patients were under home medical therapy due to their comorbidities (e.g., ACE inhibitors for hypertension treatment). No significative differences were detected among the other respiratory parameters between survivor and non-survivor subjects.

### 3.2. Untargeted Metabolomic Analysis

The peak tables resulting from the ESI+ and ESI− analyses were processed using multi-level SO-CovSel-PLSDA to identify metabolites which could be associated with the time progress of the disease. The classification problem was formulated as a three-category problem, with each class corresponding to a time point, and the overall strategy was validated using a rDCV scheme with 50 runs, with ten cancelation groups in the outer loop and eight in the inner loop. The model showed an almost perfect classification accuracy (98 ± 3%) with comparable sensitivity for all the categories. From a chemical standpoint, variable selection across the different iterations of the rDCV procedure showed a high level of consistency, with nine (ESI+) and twelve (ESI−) variables being included in most of the models. As a further confirmation, a PLS–DA model including these 21 metabolites was built and validated using the same rDCV procedure, leading to comparable values of all the classification figures of merit (accuracy and sensitivity for all the classes equal to (99 ± 1%). The nine (ESI+) plus twelve (ESI–) annotated metabolites arising from the untargeted metabolomics data’s statistical analysis are reported in Table S1. The statistical treatment of the untargeted datasets allowed the obtainment of a pool of 21 candidate biomarkers, comprising several phospholipids (compounds **8**, **16**, **17**, and **19**, Table S1), steroids (compounds **5**, **6**, **15**, and **18**), other lipids and lipid derivatives (compounds **9** and **13**), nucleobases and nucleosides (compounds **1** and **3**), other nitrogen compounds (compounds **2**, **4**, and **10**), sulfate and glucuronide conjugates (compounds **11**, **12**, and **14**), and bile acids (compounds **7**, **19**, and **21**). BA profiles were carefully evaluated in a further target metabolomic analysis.

### 3.3. Untargeted and Targeted Metabolomics Comparison

**Figure 1:**
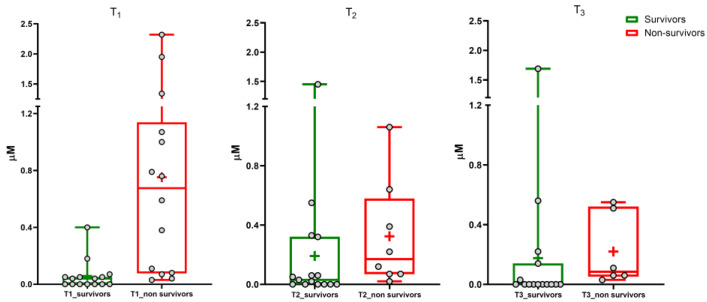
GLCA-3S. Median (line), mean (+), and the minimum and maximum values at three sampling times (T1, T2, T3).

**Figure 2:**
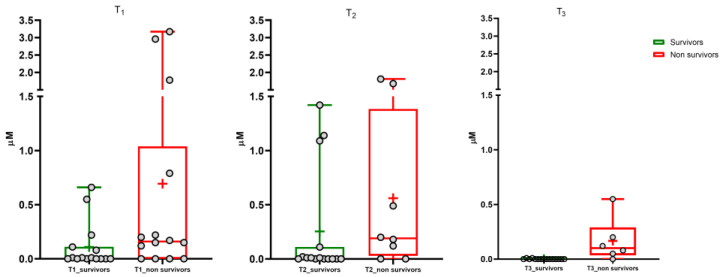
GCDCA-3S. Median (line), mean (+), and the minimum and maximum values at three sampling times (T1, T2, T3).

Data normalization was performed to compare the results for untargeted and targeted metabolomics, i.e., ion counts and micromolar concentrations, respectively. Normalization was performed by considering 0% as the lowest value in the dataset and 100% as the highest value. Figures S2 and S3 in the Supplementary Materials section shows the results for the BA of each patient. These results confirmed that we obtained similar results in the same cohort of patients by using different approaches. The untargeted and targeted metabolomics (results reported in Table S4) highlighted a significant increase in serum concentration of glycine-conjugated sulfate metabolites of chenodeoxycholic (GCDCA-3S) and lithocholic BA (GLCA-3S) during the disease. The targeted metabolomics was performed by including non-survivor patients (Table S5 in Supplementary Materials). Box and whisker plots (Figure 1 and Figure 2) show the results at the three time points for GLCA-3S and GCDCA-3S between survivors and non-survivors.

A small number of samples were available at the second and third time points due to the deaths of several patients with COVID-19 during the study. Furthermore, it must be considered that patients received parental feeding, which involves a change in BA pool dynamics, composition, and metabolism. Consequently, this paper considers mainly BA levels at T1, where the BA pool is not altered by pharmacology therapy and food intake.

The Mann–Whitney test showed a highly significant difference between survivors and non-survivors for serum GLCA-3S concentration (*p*-value < 0.0001). No significant difference was determined for GCDCA-3S (*p*-value 0.06). We also evaluated the ratios between the concentration of each sulfate metabolite and the metabolic precursor GCDCA (calculated as [BA-S metabolite]/[BA-S metabolite+GCDCA]) in order to consider the conversion from GCDCA to sulfate metabolites by the liver (Figures S4 and S5 in Supplementary Materials). The Mann–Whitney test showed a significant difference for GLCA-3S and GCDCA-3S (*p*-value < 0.001 and 0.03, respectively). A significant difference was not found for both TCDCA and GCDCA (Figure S6 in Supplementary Materials) between the two groups (*p*-value 0.22 and 0.06, respectively) and also for the other investigated BAs (*p*-value > 0.05).

### 3.4. Correlation Matrix, Logistic Regression, and ROC

The correlation between all clinical and biochemical parameters and serum BA composition was performed using Spearman’s coefficient (Supplementary Materials Table S6). Categorical variables were summarized in terms of numbers. We have also introduced the ratios between the concentrations of the BA-S and the main metabolic precursor, GCDCA.

Logistic regression was used to better assess the association between BA-S concentrations and mortality. Increases in GLCA-3S and GCDCA-3S were associated with incident mortality, as well as the ratios with the metabolic precursor. The OR (95% CI) was 26 (3.68–183.42) for GLCA-3S, 42 (5.1–345.1) for its ratio with GCDCA, 10.8 (1.96–59.83) for GCDCA-3S, and 37.5 (3.64–386.51) for its ratio with GCDCA (*p*-value< 0.01). Table S7 in the Supplementary Materials section provides the results obtained.

**Figure 3:**
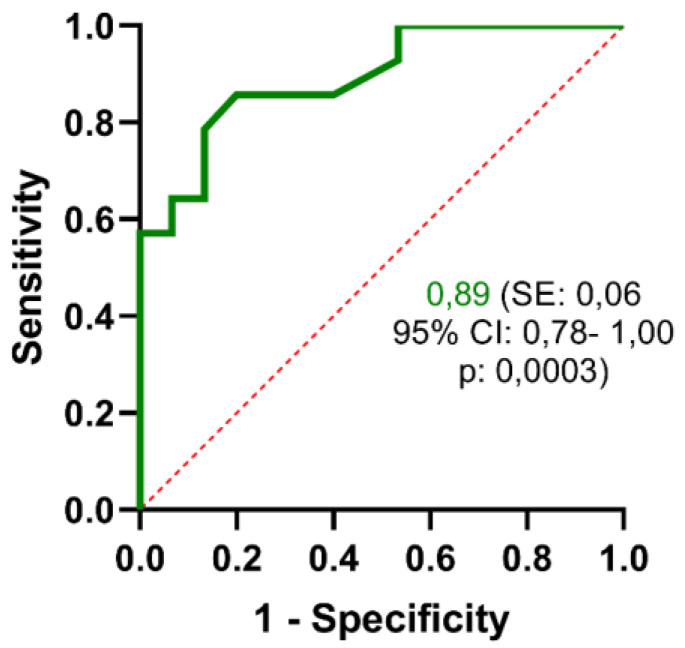
ROC curve analysis of serum GLCA-3S concentration at T1 showing area under the curve (AUC) with standard error, 95% confidence interval, and its associated *p*-value.

The GLCA-3S predictive performance was confirmed by the receiver operating characteristic (ROC) curve (Figure 3).

The area under the curve (AUC) was 0.89 (standard error 0.06). Based on the ROC curve and predictive value of the different cut-off points, the optimal cut-off point was 0.06 µM. The specificity and sensitivity values of GLCA-3S were 80.0% and 85.7%, respectively.

Thus, our data showed that high serum concentrations of BA-S, especially GLCA-3S, are associated with a worse prognosis for the patient.

### 3.5. Chemometric Analysis

**Figure 4:**
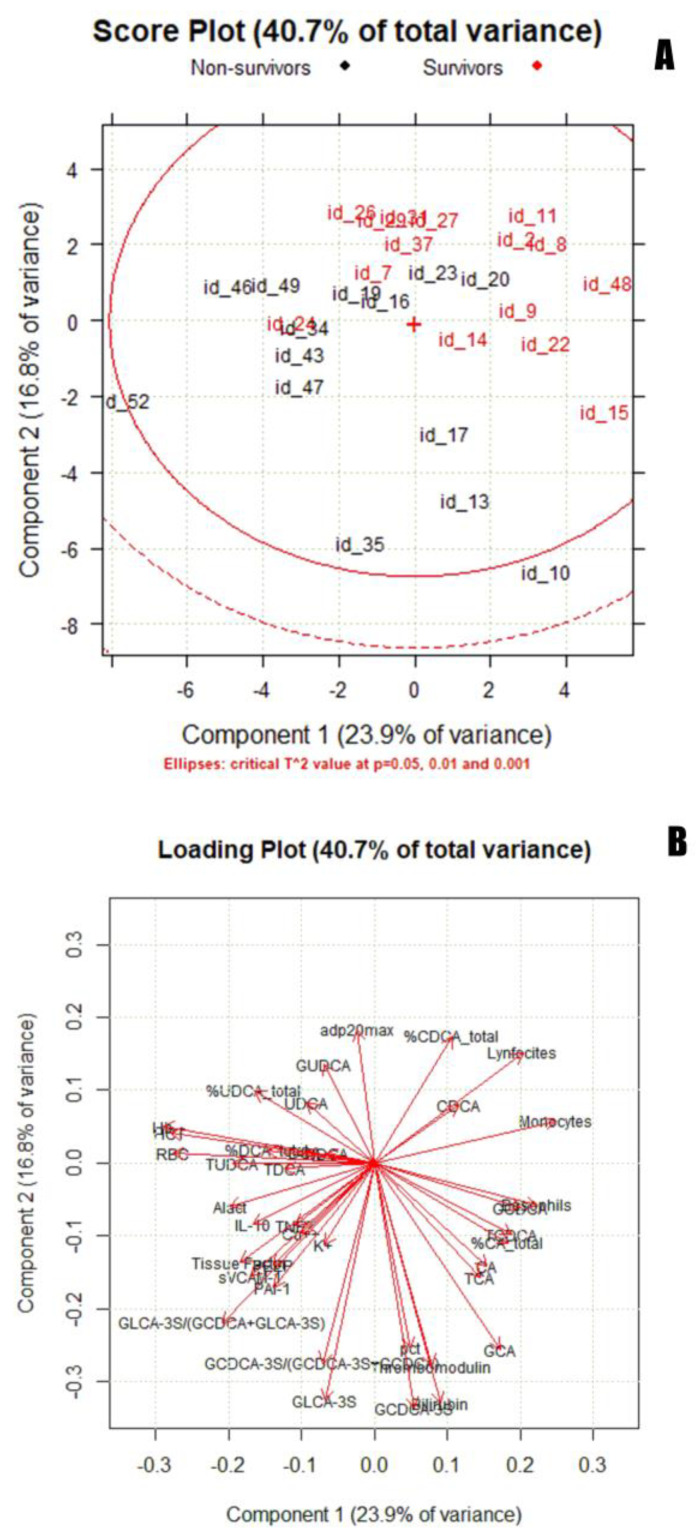
Score plot (**A**) and loading plot (**B**) for PC1 vs. PC2.

Principal component analysis (PCA) was used to identify a multidimensional space cluster within the dataset and determine the presence of outliers. Figure 4 shows a score plot (a) and loading plot (b) (PC1 vs. PC2). Figure S7 in the Supplementary Materials section shows the score plot and loading plot for PC2 vs. PC3.

The inclusion of all points in the Hottelling ellipse (Hotteling T2) indicates that the two groups belong to the same distribution (COVID-19-positive). Among COVID-19-positive subjects, the separation between survivors (red) and non-survivors (black) can be observed in the score plot along the PC2 component. Although some samples are overlapping between the two groups, survivors are placed along the positive PC2, whereas non-survivors are clustered along the negative PC2. An influence plot (Figure S8 in the Supplementary Materials section), performed using six PCs, was used to identify outliers in the data set. Two patients, one survivor and one non-survivor, had a Q value above the predefined threshold (*p* = 0.05). Loading plots allowed the identification of the discriminating variables in the explored space, which in this case were serums GLCA-3S and GCDCA-3S (loadings of −0.33 and −0.34, respectively).

## 4. Discussion

The agreement between the results of two independent mass spectrometry analyses provides solid evidence of the validity and accuracy of the experimental approach. Furthermore, targeted metabolomics found a difference in serum GLCA-3S between survivors and non-survivors in a larger cohort of patients with early-stage COVID-19. According to our results, in the non-survivor group, a greater amount of GCDCA could be converted to sulfate metabolites. To the best of our knowledge, there are few studies in which BA-S has been evaluated in serum of healthy subjects. Physiological values have been previously reported [14,24] as 275.5 ± 15.0 nM and 76.34 ± 53.9 nM for GLCA-3S, and 247.8 ± 12.9 nM for GCDCA-3S. The GLCA-3S median of our serum concentrations at T1 of 675 nM (80–1140) was higher in non-survivor subjects than in the physiological conditions reported in the literature. The same result was established for GCDCA-3S, where the median was 0.16 µM (0.00–1.04).

Statistical and chemometrics analyses were based on our data for serum BA concentrations and different clinical and biochemical function tests reported in the ATTAC-CO project (ClinicalTrials.gov Identifier: NCT04343053). Logistic regression highlighted the significant correlation between mortality and BA-S, specifically for GLCA-3S. Finally, a multivariate approach was performed using serum BA concentrations as the most significant variables of the correlation matrix and the most recognized biomarkers related to enterohepatic dysfunction and an inflammation state [2]. GLCA-3S has also been established in the PCA as a powerful biomarker for COVID-19 severity in the early stage of the disease.

Finally, we investigated each biochemical parameter correlated with BA in the matrix reported in the Supplementary Material (Table S6) and their potential involvement in several physiological pathways and tissues. The table below highlights the most interesting results from the literature search, stratified by tissue or organ (Table 2).

First, it is necessary to underline that an increase in serum GLCA-3S and GCDCA-3S does not necessarily follow a significant increase in total serum BA (Figure S9 in Supplementary Materials) (*p*-value 0.56). Thus, the increase of serum GLCA-3S and GCDCA-3S is independent of any potential liver dysfunction such as cholestasis (as confirmed by the physiological levels of bilirubin and other biomarkers), where total BA concentration is at least 10–20 times higher. Considering non-hepatic damage, another association between BA-S has been found in the literature. Specifically, GLCA-3S and uncommon glycocholenate sulfate (possibly synthesized from glycine-amidation and the sulfation of 3-beta-hydroxy-5-cholenoic acid) were associated with an increased incidence of atrial fibrillation (AF) [39]. This correlation was independent of other risk factors for AF, including kidney function, alcohol consumption, and markers of liver damage. Notably, other factors were involved in the coagulation cascade correlated with BA-S, such as plasminogen activator inhibitor-1 (PAI-1), thrombomodulin, and tissue factors. All these results seem to support that serum BA-S could be positively correlated with an increased incidence of AF and more generally, cardiovascular disease.

Moreover, the infection caused by the SARS-CoV-2 virus has a relatively high risk of sepsis and multiple organ failure [40]. A recent retrospective pilot study has shown that despite COVID-19 patients meeting SEPSIS-3 criteria, phenotypes of dysregulated host responses following infection by bacteria or SARS-CoV-2 appear to be substantially different [41]. In previous studies, high values of total BA-S, TCDCA-3S, and GCDCA-3S have been found in non-surviving patients with community-acquired pneumonia and sepsis in the control group [42]. It was hypothesized to be related to early-stage cholestasis in patients with sepsis. On the contrary, we found no indications of liver damage and cholestasis in the studied patients, while we identified a positive correlation between serum BA-S and serum pct (*p*-value < 0.05, see Table S4 in Supplementary Materials). Pct is one marker used for sepsis detection [27], in which the peculiar characteristic is the so-called “cytokine storm”, a potentially fatal immune reaction [28]. Not surprisingly, we found a positive correlation between BA-S and parameters related to patients' immune systems, especially TNF-α and s-VCAM-1, an endothelial biomarker which plays a key role in inflammation and immune responses. Notably, s-VCAM-1 was also identified as the only marker significantly associated with the long-term risk of AF [32].

Viral load has been correlated with COVID-19 mortality [43]. Further studies could confirm the association between viral load and BA, also considering the BA effect on ACE2 expression, the receptor through which SARS-CoV-2 enters human cells.

Generally, it is important to specify that the biological explanation of altered levels of BA-S in disease is still unclear [44]. Several lines of evidence support the role of nuclear receptors in maintaining BA homeostasis by regulating Sult2a1 expression. However, there is limited functional data to confirm that this regulation leads to significant changes in BA–S concentrations in hepatic or extrahepatic tissues and fluids. Renal clearance is certainly the primary factor responsible for regulating BA-S levels in blood. On the other hand, our patients did not show any alterations in renal function at the inclusion time, therefore the higher levels of these species in peripheral blood cannot be attributed to this. The explanation is likely due to increased sulfation at the hepatic level. Anyway, the mechanism behind this dysbiosis remains unclear and will need further investigation.

Regarding the link between BAsand inflammatory processes, it is particularly interesting to note the findings reported by Duboc et al. [45]. Reduced desulfation in inflammatory bowel diseases, with a consequent accumulation of sulfated species, leads to a logical reduction in secondary BA, which are key mediators of the anti-inflammatory activity of BA on the TGR5 receptor.

## 5. Conclusions

BA-S metabolites in serum have been identified as a potential clinical biomarker in a cohort of survivors of COVID-19 using two different mass spectrometry approaches, namely untargeted and targeted metabolomics. Thus, the targeted analytical method for serum BA quantification was applied to a larger cohort of patients with COVID-19. Among all the investigated biomolecules and variables, GLCA-3S was identified as the main clinical biomarker correlated with mortality in the early stage of the illness, suggesting its use as an indicator of disease severity in the early stage of COVID-19.

AF and cardiovascular complications have been recognized among the leading causes of death in patients with COVID-19 in the literature [46]. Based on correlation coefficients, our findings on BA-S agree with the biomarkers reported in the literature regarding AF [39]. Again, our data suggest the concept that the involvement of serum BA-S in sepsis pathology should not only be related to early cholestasis, as previously reported [42].

Vaccines certainly have the ability to reduce the onset of COVID-19 symptoms; however, they do not preclude the use of BAs as disease severity biomarkers in clinical practice, as seen with other molecules [47]. According to our results, BA-S would be a powerful and valuable biomolecule when a “precision medicine” approach is required to personalize the therapy of COVID-19 patients appropriately.

## Figures and Tables

**Table 1:** Baseline characteristics in survivors versus non-survivors. Data are reported as numbers (percentages) or mean ± standard deviationas appropriate. *p*-value: for the comparison between survivors vs. non-survivors. BMI: body mass.

	Survivors (*n* = 15)	Non-Survivors (*n* = 15)	*p*-Value (α: 0.05)
Age (years)	61 ± 9	71 ± 8	0.04
Male sex, no. (%)	12 (80)	12 (80)	˃0.99
BMI (kg/m^2^)	28 ± 4	28 ± 4	˃0.99
Comorbidities, no. (%)			
Hypertension	7 (47)	11 (73)	0.15
Dyslipidemia	2 (13)	4 (27)	0.65
Former smoker	2 (13)	7 (47)	0.11
Diabetes type 2	3 (20)	2 (13)	˃0.99
Prior MI	0 (0)	2 (13)	0.48
Prior coronary revascularization	0 (0)	2 (13)	0.48
Prior CVA	0.07 (0.2)	0 (0)	0.33
Peripheral artery disease	1 (7)	5 (33)	0.17
Chronic coronary syndrome	0 (0)	2 (13)	0.48
Transient ischemic attack (mini stroke)	1 (7)	0 (0)	˃0.99
Atrial flutter	2 (13)	2 (13)	˃0.99
CHA₂DS₂-VASc score for atrial fibrillation stroke risk (≥ 2)	6 (40)	11 (73)	0.14
Other CVD	1 (7)	1 (7)	˃0.99
Chronic kidney disease	3 (20)	8 (53)	0.13
Cancer	2 (13)	4 (27)	0.65
Home medical therapy, no. (%)			
Aspirin	0 (0)	3 (20)	0.22
Statins	2 (13)	5 (33)	0.39
ACE inhibitors	4 (27)	9 (60)	0.26
Beta-blockers	2 (13)	5 (33)	0.39
Calcium channel blockers	2 (13)	5 (33)	0.39
Diuretics	1 (7)	5 (33)	0.17
Glucose drugs	1 (7)	1 (7)	˃0.99

**Table 2:** Clinical parameters correlated with BA-S and their possible implication in different pathways involved in COVID-19 according to the published literature.

System	Clinical Parameters	Correlation with	Reported in the Literature
Immune system	Lymphocytes	GLCA-3S	Lymphopenia has been defined as an effective and reliable indicator of the severity and hospitalization in patients with COVID-19 [26].
		GLCA-3S/(GCDCA + GLCA-3S)	
	Procalcitonin (PCT)	GLCA-3S	Pct is one marker used for sepsis detection and is a sensitive marker of acute COVID-19 disease [2,27].
		GCDCA-3S	
		GCDCA-3S/(GCDCA-3S+GCDCA)	
	Interleukin 10 (IL-10)	GLCA-3S	Increased expression of IL-10 in patients who have progressed to severe or life-threatening COVID-19 disease [4]. (Contoli et al. 2021).
		GLCA-3S/(GCDCA+GLCA-3S)	
	Tumor necrosis factor alpha (TNF-α)	GCDCA-3S/ (GCDCA+GCDCA-3S)	Cytokine storm, characterized by an increased production of cytokines, mainly IL-6, C-reactive protein (CRP), TNF-α, IL-1β, IL-33, IFNγ, and others, is a phenomenon noticed in patients with severe forms of COVID-19 [28]. This process fuels the inflammation that characterizes pneumonia due to a defect in the control system that can no longer block the immune response.
Cardiovascular system	Tissue factor (TF)	GLCA-3S/(GCDCA+GLCA-3S)	TF has been associated with developing thrombotic phenomena in COVID-19, which often causes mortality [29].
	Thrombomodulin	GCDCA-3S	
	Plasminogen activator inhibitor-1 (PAI-1)	GLCA-3S	PAI-1, the major physiological inhibitor of tissue-type plasminogen activators in serum, was significantly associated with stroke in AF [30]. There is epidemiological evidence that PAI-1 may contribute to the development of ischemic cardiovascular disease [31].
		GLCA-3S/(GCDCA+GLCA-3S)	
	Soluble vascular cell adhesion molecule-1 (s-VCAM)	GLCA-3S/(GCDCA+GLCA-3S)	sVCAM-1 was identified as the only marker of inflammation significantly associated with long-term risk of AF among the other 13 biomarkers investigated [32].
	Lactic acid	GLCA-3S	It is known that serum lactic acid levels get higher when strenuous exercise or other conditions—such as heart failure, a severe infection (sepsis), or shock—lower blood flow and oxygen throughout the body [33].
		GLCA-3S/(GCDCA+GLCA-3S)	
Kidney	Potassium	GCDCA-3S	Recent studies have reported a high prevalence of electrolyte disorders in patients with SARS-CoV-2 infection, including sodium, potassium, chloride, and calcium abnormalities [34,35,36,37]. However, electrolyte disorder is also positively related to severe clinical outcomes, such as cardiovascular diseases [38].
		GCDCA-3S/(GCDCA-3S+GCDCA)	
	Calcium	GLCA-3S/(GCDCA+GLCA-3S)	

## Data Availability

The data presented in this study are available upon request from the corresponding author.

## Supplementary Materials

The following supporting information can be downloaded at: https://www.mdpi.com/article/10.3390/cells13181576/s1, Untargeted metabolomics workflow: LC–MS conditions, data pre-processing, statistical analysis, and compound annotation; Table S1. List of the 21 annotated metabolites following MS/MS spectra inspection of the variables selected from the statistical analysis of the untargeted metabolomics datasets related to the untargeted metabolomic analysis; Figure S1. MS/MS spectra of compounds 19 (a) and 21 (b) that were tentatively identified as BA sulfoglycolithocholic acid (GLCA-3S) and sulfoglycochenodeoxycholic acid (GCDCA-3S) after spectra inspection related to the untargeted metabolomic analysis. Targeted metabolomics workflow: sample preparation, LC–MS conditions; Table S2 MS/MS transitions, collision energy, and cone of every single compound. Targeted metabolomics statistical analysis; Table S3. Clinical and biochemical parameters in survivors versus non-survivors at inclusion. Data are reported as percentage, mean ± standard deviation, or median [interquartile range] as appropriate. P-value: for the comparison between survivors vs. non-survivors. WBC: white blood cells. mcL: million per microliter. U/L: units per liter. FEU: fibrinogen equivalent unit.; Table S4. Untargeted metabolomics results for GLCA-3S and GCDCA-3S stratified by patient (at T1, T2, T3); Figure S2. Serum GCDCA-3S comparison between untargeted and targeted metabolomics for each patient; Figure S3. Serum GLCA-3S comparison between the untargeted and targeted metabolomics for each patient; Table S5. Bile acid concentration in survivor and non-survivor patients (mean ± standard deviation). From the total database, we exclude one non-survivor who had colon cancer; Figure S4. Box and whisker plot of serum GLCA-3S/(GLCA-3S+GCDCA) in survivors and non-survivors at three sampling times. Median (line), mean (+), and minimum and maximum values; Figure S5. Box and whisker plot of serum GCDCA-3S/(GCDCA-3S+GCDCA) in survivors and non-survivors at three sampling times. Median (line), mean (+), and minimum and maximum values; Figure S6. GCDCA, TCDCA, GCDCA-3S, and GLCA-3S concentrations in survivors and non-survivors at T1. Median (line), mean (+), and minimum and maximum values; Table S6. Results of correlation matrix. Correlation coefficients and P-value; Table S7. Logistic regression results for the association between BA-S concentrations and mortality; Figure S7. Score plot (a) and loading plot (b) for PC2 vs. PC3; Figure S8. Influence plot performed using six principal components; Figure S9. Box and whisker plot of serum total BA at T1 in survivors and non-survivors. Median (line), mean (+), and minimum and maximum values.
