# Supplementary material for: Sulfated Bile Acids in Serum as Potential Biomarkers of Disease Severity and Mortality in COVID-19
Source: Cells. 2024 Sep 19;13(18):1576. doi: 10.3390/cells13181576 (PMC11430696; doi:10.3390/cells13181576)

Sulfated Bile Acids in Serum as Potential Biomarkers of Disease Severity and Mortality in COVID-19

Emanuele Porru[1], Rossana Comito[2], Nicolò Interino[3], Andrea Cerrato[4], Marco Contoli[5], Paola Rizzo[6,7], Matteo Conti[8], Gianluca Campo[9], Savino Spadaro[10], Cristiana Caliceti[11,12,13,14], Federico Marini[4], Anna L. Capriotti[4], Aldo Laganà[4], Aldo Roda*[12,15]

1 Occupational medicine unit, Department of Medical and Surgical Sciences, Alma Mater Studiorum, University of Bologna, 40138 Bologna, Italy
2 Division of Occupational Medicine, IRCCS Azienda Ospedaliero-Universitaria di Bologna, 40138 Bologna, Italia
3 IRCCS Istituto delle Scienze Neurologiche di Bologna, 40139 Bologna, Italia
4 Department of Chemistry, "Sapienza" University of Rome, 00185- Rome, Italy
5 Respiratory Section, Department of Morphology, Surgery, and Experimental Medicine, University of Ferrara, 44121-Ferrara, Italy.
6 Department of Translational Medicine and Laboratory for Technologies of Advanced Therapies (LTTA), University of Ferrara, 44121- Ferrara, Italy
7 Maria Cecilia Hospital, GVM Care & Research, Cotignola, 48022- Ravenna, Italy
8 Department of Public Health, Local Unit of Imola, Health Service of the Emilia-Romagna Region, 40026 Imola, Italy
9 Cardiovascular Institute, Azienda Ospedaliero-Universitaria di Ferrara, 44124- Cona, Ferrara, Italy.
10 Intensive Care Unit, Department of Morphology, Surgery, and Experimental Medicine, University of Ferrara, 44121-Ferrara, Italy.
11 Department of Biomedical and Neuromotor Sciences, Alma Mater Studiorum, University of Bologna, 40123- Bologna, Italy
12 Biostructures and Biosystems National Institute (INBB), 00136- Rome, Italy
13 Interdepartmental Centre for Industrial Agrofood Research-CIRI Agrofood, University of Bologna, 47521 Cesena, Italy.
14 Interdepartmental Center of Industrial Research "CIRI"-Energy and Environment, Alma Mater Studiorum, University of Bologna, 40126 Bologna, Italy
15 Department of Chemistry "G.Ciamician", Alma Mater Studiorum- University of Bologna, 40126- Bologna, Italy

[*]**Corresponding author.**

To whom correspondence should be addressed:
Professor Emeritus Aldo Roda
e-mail: aldo.roda@unibo.it

1.1. Untargeted Metabolomics workflow

1.1.1. LC-MS conditions

A Vanquish binary pump H (Thermo Fisher Scientific, Bremen, Germany), equipped with a controlled temperature autosampler and column compartment, was used for chromatographic separation on a Luna Omega Polar C18 (100 × 2.1 mm, 1.6 µm particle size, Phenomenex, Torrance, USA). The mobile phases were H_2O/HCOOH (99.9:0.1, *v/v*; phase A) and MeOH/HCOOH (99.9:0.1, *v/v*; phase B) and were mixed with the following gradient: 1% phase B for 2 minutes; 1% phase B to 99% phase B in 15 minutes; 99% phase B for 5 minutes (washing step) and 1% phase B for 5 minutes (reconditioning step). The column was maintained at 50 °C with a constant flow of 400 µL min^{-1}. The chromatographic system was coupled to a hybrid quadrupole-Orbitrap mass spectrometer Q Exactive (Thermo Fisher Scientific) via a heated ESI source. Samples and QCs were analyzed both in positive and negative ion mode. The ESI source parameters were: capillary temperature at 220 °C and 180 °C for positive (+) and negative (-) polarity respectively, spray voltage at 3200 V (+) and 2800 V (-), auxiliary gas heater temperature at 280 °C (+) and 180 °C (-), sheath gas at 50 (arbitrary units), auxiliary gas at 25 (arbitrary units), sweep gas was 0 (arbitrary units), and S-Lens RF level was 50 (%).

Samples were run in single-MS full-scan mode, to obtain high-quality peak shapes for high- and low-abundance substances(Schrimpe-Rutledge et al. 2016). At the end of each sequence, three QC injections (identification-only QC) were run in the top five data-dependent acquisition (DDA) mode, consisting of one full-scan acquisition followed by five tandem MS scans to obtain MS/MS for subsequent feature identification. Full scan acquisition mode was performed in the range m/z 70-1000 with a resolution of 70.000 (full width at half-height, FWHM, m/z 200). The automatic gain control (AGC) target value was 500.000 in full scan, with a maximum ion injection time set at 50 ms. The isolation window width was 2 m/z. For identification-only QCs, the top 5 DDA mode was performed with the AGC target set at 100.000. Higher-energy collisional dissociation (HCD) was performed at 40 normalized collision energy with a resolution of 35.000 (FWHM m/z 200). Dynamic exclusion was set to 3 s. Before analysis, the mass spectrometer was calibrated using a calibration solution provided by the manufacturer (external calibration).

Raw MS/MS data files were acquired by Xcalibur software (version 3.1, Thermo Fisher Scientific). The column stability and performance were tested before and after each analytical section using blank samples and the external standard solutions. System optimization and conditioning, consisting of ten consecutive QC sample injections, preceded the blank sample for background subtraction. After further system reconditioning with ten more QCs samples, randomized samples and controls were run in groups of five, followed by a QC injection. Internal standards spiked in the samples were employed to rapidly check potential outliers or macroscopic damages during analysis, rather than used for sample normalization, which was later accomplished during data processing by QC-based normalization.

1.1.2. Data pre-processing

The raw data obtained from the analysis of samples, QCs, and blanks were pre-processed using the software Compound Discoverer version 3.1 (Thermo Fisher Scientific). The adaptive curve regression model obtained feature alignment; whenever the adaptive curve model failed, the linear model was automatically selected instead. Features were aligned and filtered to remove the compounds in the blank samples from the real samples and QCs. The blank sample employed for data pre-processing (after 10 QC runs) allowed to discard both the contaminants present in mobile phases and the HPLC-MS system and the compounds subjected to high carry-over effects (more than 10%), which might have altered peak areas, resulting in biased statistical analysis. QC-based normalization of the features was carried out. A linear regression of the peak areas in the QC samples over time was built for each feature. Subsequently, the linear regression was corrected so that the slope of each curve was zero (for most compounds, the linear regressions showed a negative slope that can be easily attributed to the progressive loss of ionization efficiency due to matrix effects). Eventually, the sample areas of each feature were normalized based on their trend over time. Before statistical analysis, the features were filtered to remove all compounds that were not present in all QC samples and those whose area in the QCs presented a standard deviation higher than 25%. The remaining features associated with an MS/MS spectrum in the *identification-only* QC sample runs were exported for statistical analysis.

1.1.3. Statistical Analysis and compound annotation untargeted metabolomics

Biomarker identification based on untargeted metabolomics was performed through a chemometric classification strategy based on coupling multilevel partial least squares discriminant analysis (ML-PLSDA), feature reduction by means of the covariance selection (CovSel) algorithm and repeated double cross-validation (rDCV). More specifically, ML-PLSDA exploits the variance partitioning scheme of repeated measurements ANOVA to remove the inter-subject variability prior to performing PLS-DA modeling. In detail, each of the two data matrices resulting from ESI+ and ESI-

analyses was split into two submatrices, one collecting the average profiles of the patients (i.e., the mean values of the features across the three time points) and the other one (which is the one that was subsequently subjected to PLS-DA analysis) accounting for the within-subject variation and obtained by centering the feature vectors collected on each individual around the corresponding patient's mean profile). The resulting ESI+ and ESI- within-subject submatrices were then joined to constitute a multi-block data set which was processed by a technique allowing at the same time to perform variable selection (and, therefore, identifying putative markers), namely Sequential and Orthogonalized Covariance Selection (SO-CovSel) coupled to PLS-DA. CovSel is a variable selection technique providing the minimum set of non-redundant predictors and, therefore, a very parsimonious list of candidate metabolites. SO-CovSel allows its generalization to the case where more than a single block of predictors is available. In these cases, at first, CovSel is applied to the first block, resulting in a set of selected variables. The second block is orthogonalized with respect to these selected variables to avoid redundancies, and lastly, CovSel is applied again to the orthogonalized second block, to extract other predictors. Eventually, a PLS-DA model was calculated on the data resulting from the concatenation of the variables selected from the ESI- and ESI+ block.

The reliability of the overall strategy was assessed through a repeated double cross-validation (rDCV) approach. rDCV consists of two loops of cross-validation nested in one another: the outer loop mimicking the presence of an external test set and being used only to test the models built at each iteration, and the inner loop which constitutes the basis for model building and model selection. The procedure is repeated a sufficient number of times (here 50), after changing the distribution of the samples across the cancelation groups, to rule out the possibility that the results be only due to a specific splitting scheme. Moreover, repetition allows to calculate confidence intervals for the classification figures of merit and the model parameters.

The selected variables were then annotated by inspection of their MS/MS spectra. For metabolites present in the mzCloud database, spectra matching was automatically performed by Compound Discoverer software. All other metabolites were tentatively identified by matching the experimental MS and MS/MS spectra to available spectral libraries, spectra reported in the literature, and the predicted spectra reported in the Human Metabolome Database (HMDB)(Wishart et al. 2007). The list of the annotated metabolites is reported in Table S1.

Table S1. List of the 21 annotated metabolites following MS/MS spectra inspection of the variables selected from the statistical analysis of the untargeted metabolomics datasets, related to Untargeted metabolomic analysis

	Tentative identification	Molecular Formula	Molecular Weight	RT (min)	Data Matrix	
1	Guanine	$C_5H_5N_5O$	151.0495	1.9	ESI (+)	T1 vs T2 vs T3
2	4-Anilino-4-oxobutanoic acid	$C_{10}H_{11}NO_3$	193.0740	9.2	ESI (+)	T1 vs T2
3	Dihydrouridine	$C_9H_{14}N_2O_6$	246.0849	0.9	ESI (+)	T1 vs T2 vs T3
4	Aspartyl-phenylalanine	$C_{13}H_{16}N_2O_5$	280.1056	4.1	ESI (+)	T2 vs T3
5	Cholesterol isomer	$C_{27}H_{46}O$	386.3549	20.3	ESI (+)	T1 vs T3
6	Setariol isomer	$C_{27}H_{42}O_3$	414.3134	16.0	ESI (+)	T2 vs T3
7	Hydroxycholenoylglycine isomer	$C_{26}H_{41}NO_4$	431.3032	13.9	ESI (+)	T1 vs T2 vs T3
8	Oleyl sphingomyelin	$C_{41}H_{81}N_2O_6P$	728.5827	21.0	ESI (+)	T1 vs T2
9	TG (16:0/18:1/20:4)	$C_{57}H_{100}O_6$	880.7483	19.1	ESI (+)	T1 vs T2 vs T3
10	4-Pyridoxic acid	$C_8H_9NO_4$	183.0532	2.1	ESI (-)	T2 vs T3
11	Sulfooxy-phenylacetic acid	$C_8H_8O_6S$	232.0043	2.8	ESI (-)	T1 vs T2 vs T3
12	Hydroxyindolacetyl sulfate	$C_{10}H_9NO_6S$	271.0153	3.7	ESI (-)	T1 vs T2 vs T3
13	Palmitoyl glycine	$C_{18}H_{25}NO_3$	313.2624	16.5	ESI (-)	T1 vs T2 vs T3
14	Vanillyl alcohol glucuronide	$C_{14}H_{18}O_9$	330.0958	3.5	ESI (-)	T1 vs T2 vs T3
15	Androsterone sulfate isomer	$C_{19}H_{30}O_5S$	370.1818	12.4	ESI (-)	T1 vs T2 vs T3
16	LPE O-16:0	$C_{21}H_{46}NO_6P$	439.3069	17.0	ESI (-)	T1 vs T2 vs T3

17	LPE 16:0	$C_{21}H_{44}NO_7P$	453.2857	16.7	ESI (-)	T1 vs T2 vs T3
18	Androsterone glucuronide isomer	$C_{25}H_{38}O_8$	466.2568	13.9	ESI (-)	T1 vs T2
19	Sulfoglycolithocholic acid	$C_{26}H_{43}NO_7S$	513.2759	14.9	ESI (-)	T2 vs T3
20	LPE 22:5	$C_{27}H_{46}NO_7P$	527.3016	16.6	ESI (-)	T1 vs T2 vs T3
21	Sulfoglycochenodeoxycholic acid	$C_{26}H_{43}NO_8S$	529.2706	14.2	ESI (-)	T2 vs T3

Figure S1. MS/MS spectra of compounds 19 (a) and 21 (b) that were tentatively identified as BA sulfoglycolithocholic acid (GLCA-3S) and sulfoglycochenodeoxycholic acid (GCDCA-3S) after spectra inspection, related to untargeted metabolomic analysis

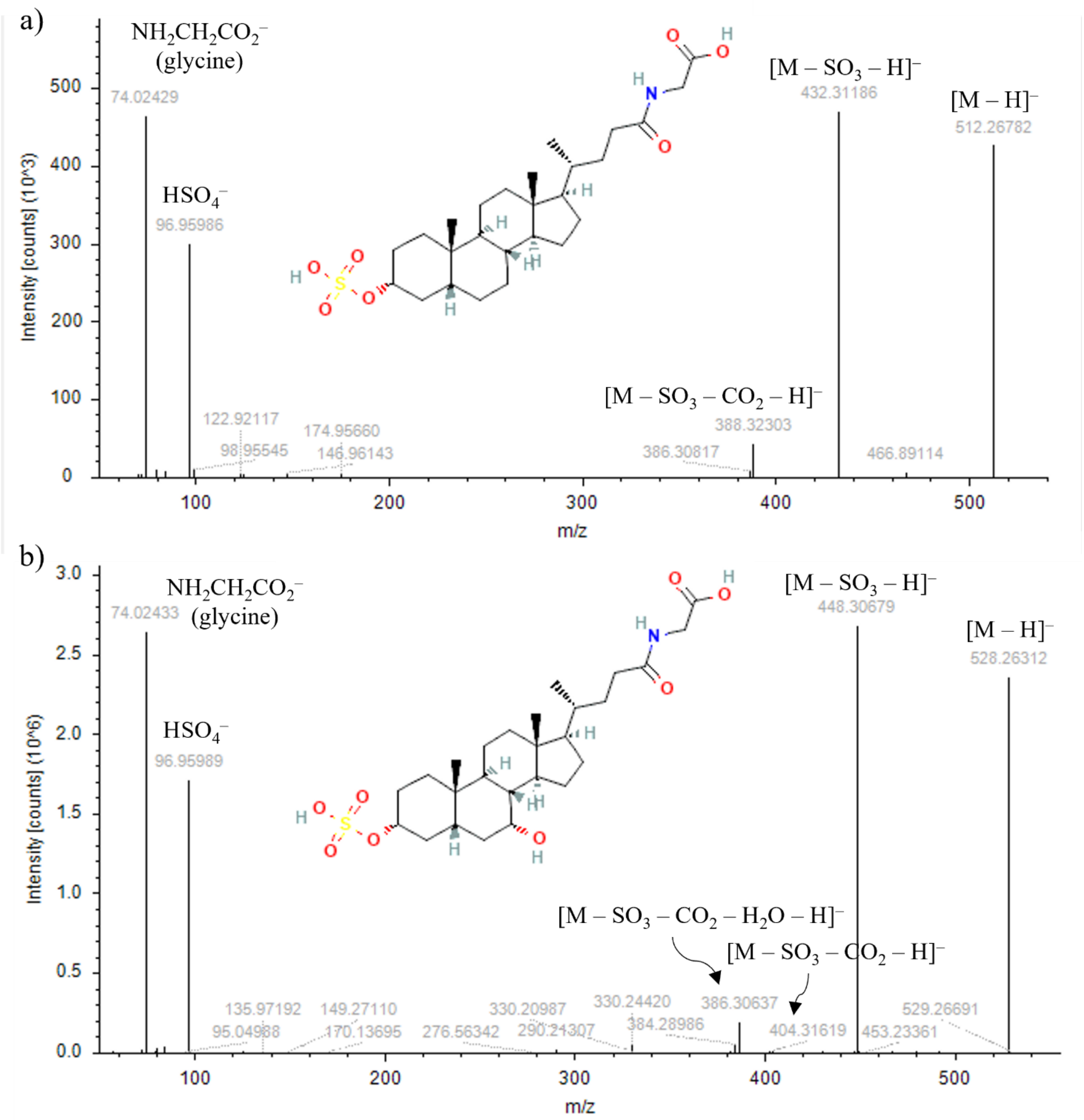

1.2. Targeted Metabolomics workflow

1.2.1. Sample preparation

Pooled drug-free human serum was purified using activated charcoal to remove endogenous BA. 400 mL of the serum was mixed with 20 g of activated charcoal and the mixture was shaken moderately on an orbital shaker overnight (about 17 h) at room temperature. After centrifugation at 19,500 rpm for 1h, the supernatant of purified serum was transferred to clean tubes and kept at −80 ◦C until use.

For BA extraction with SPE, the column was conditioned with 5 mL of MeOH and 5 mL of H2O before sample loading. Serum samples were loaded into the conditioned column, washed with 5 ml of H2O, and then eluted with 5 ml methanol. The eluate was dried under vacuum and then reconstituted with 100 µL of 65% ammonium acetate buffer 15 mM at pH 8.00 and 35% ACN: MeOH 75:25 v/v and injected into the HPLC-ESI-MS system.

1.2.2. LC-MS conditions

Liquid chromatography was performed using a 2690 Alliance system (Waters, Milford, MA, USA). Analytical separation was achieved using a XSelect CSH Phenyl-Hexyl (5 µm, 150 mm × 2.1 mm i.d, Waters) column kept at a constant temperature of 20°C through-out the analyses.

The mobile phase was constituted by HPLC grade water with 15 mM ammonium acetate buffer at pH 8.00 (A component) and acetonitrile: methanol 75:25 v/v (B component).

Final separation was achieved at 0.15 mL/min flow rate under gradient elution conditions: 35% B for 10 min, 35-50% B from 10 to 10.30 min, 50% from 10.30 to 17 min, 50–90% B from 17 min to 17.30 min and 90% B from 17.30 min to 24 min. Re-equilibration at 35% B between analyses was achieved in 21 min, for a total run time of 45 min. The injected sample volume was 10µL. The autosampler temperature was kept at a temperature of 7°C. Table 2 summarizes the retention time of each analyte.

Analyses were performed with a triple quadruple mass spectrometer (Quattro-LC, Micromass) operating in the multiple reaction monitoring (MRM) acquisition mode. The data were managed and processed using MassLinx V4.0 software (Waters).

The MS/MS experimental conditions of all analytes included in the method were tuned by direct infusion. The MS/MS transitions specific to each compound were monitored for quantification. Nitrogen was used as nebulizer gas at 120 L/h flow rate and as desolvation gas at 587 L/h. Ion source block and desolvation temperatures were set at 130 °C and 180 °C, respectively. Capillary voltage was 2.7 kV.

Table S2. MS/MS transitions, collision energy, and cone of every single compound.

BA	RETENTION TIME (min)	TRANSITION	COLLINION ENERGY (eV)	CONE (Volts)
Glyco- 3-sulfate,7α-hydroxy-5β-cholan-24-oic acid (GCDCA-3S)	4.19	[528.26] > [448.31]<br>[528.26] > [528.26]	40<br>15	50<br>50
3α,7β-dihydroxy-5β-cholan-24-oic acid (UDCA)	5.09	[391.17] > [391.17]<br>[391.17] > [323.21]	20<br>30	60<br>60
Glyco-3α,7β-dihydroxy-5β-cholan-24-oic acid (GUDCA)	5.51	[448.24] > [74.06]<br>[448.24] > [448.24]	40<br>15	45<br>40
3-sulfate-5β-cholan-24-oic acid (LCA-3S)	5.57	[455.33] > [455.33]<br>[455.33] > [97.0]	40<br>60	55<br>65
3α,7α,12α-trihydroxy-5β-cholan-24-oic acid (CA)	5.73	[407.12] > [407.12]<br>[407.12] > [343.20]	15<br>45	60<br>75

Glyco-3-sulfate-5β-cholan-24-oic acid (GLCA-3S)	5.80	[512.47] > [431.94]<br>[512.47] > [512.47]	30<br>15	30<br>30
Glyco- 3α,7α,12α-trihydroxy-5β-cholan-24-oic acid (GCA)	6.25	[464.31] > [74.06]<br>[464.31] > [464.31]	40<br>15	50<br>40
Tauro-3α,7β-dihydroxy-5β-cholan-24-oic acid (TUDCA)	6.35	[498.23] > [123.75]<br>[498.23] > [80.0]	50<br>80	85<br>90
Tauro-3α,7α,12α-trihydroxy-5β-cholan-24-oic acid (TCA)	7.12	[514.17] > [79.89]<br>[514.17] > [482.2]	70<br>40	75<br>50
3α,7α-dihydroxy-5β-cholan-24-oic acid (CDCA)	10.78	[391.17] > [373.2]<br>[391.17] > [323.2]	35<br>40	60<br>60
Glyco-3α,7α-dihydroxy-5β-cholan-24-oic acid (GCDCA)	12.21	[448.3] > [73.96]<br>[448.3] > [448.3]	40<br>15	60<br>30
3α,12α-dihydroxy-5β-cholan-24-oic acid (DCA)	13.15	[391.17] > [345.12]<br>[391.17] > [391.17]	35<br>15	60<br>60
Glyco-3α,12α-dihydroxy-5β-cholan-24-oic acid (GDCA)	15.05	[448.3] > [73.96]<br>[448.3] > [448.3]	40<br>15	60<br>30
Tauro- 3α,7α-dihydroxy-5β-cholan-24-oic acid (TCDCA)	15.22	[498.3] > [123.7]<br>[498.23] > [80.0]	50<br>80	85<br>90
Tauro-3α,12α-dihydroxy-5β-cholan-24-oic acid (TDCA)	18.45	[498.3] > [123.7]<br>[498.23] > [80.0]	50<br>80	85<br>90
Glyco-3α-hydroxy-5β-cholan-24-oic acid (GLCA)	21.64	[432.3] > [73.93]<br>[432.3] > [432.3]	30<br>15	60<br>45
3α-hydroxy-5β-cholan-24-oic acid (LCA)	21.75	[375.23] > [375.23]<br>[375.23] > [357.2]	20<br>40	60<br>60
Tauro-3α-hydroxy-5β-cholan-24-oic acid (TLCA)	22.66	[482.15] > [79.89]<br>[482.15] > [482.15]	60<br>20	85<br>70
Internal Standards				
Ursodeoxycholic acid-d4	5.09	[395.3] > [395.3]	20	60
Cholic acid-d4	5.73	[411.3] > [411.3]	15	60
Glycocholic acid-d4	6.25	[468.4] > [74.0]	40	50
Chenodeoxycholic acid-d4	10.78	[395.3] > [395.3]	15	30
Glycochenodeoxycholic acid-d4	12.21	[452.3] > [74.0]	40	60
Deoxycholic acid-d4	13.15	[395.3] > [395.3]	15	60
Taurochenodeoxycholic acid-d4	15.22	[502.3] > [502.3]	15	40
Lithocholic acid-d4	21.75	[379.3] > [379.3]	40	60

Nitrogen was used as nebulizer gas at 120 L/h flow rate and as desolvation gas at 587 L/h. Ion source block and desolvation temperatures were set at 130 °C and 180 °C, respectively. Capillary and cone voltages were 2.7 kV and 50 V, respectively.

1.2.3. Targeted metabolomics statistical analysis

Principal component analysis (PCA) was performed to visualize the clustering of the two groups of patients (survivor and no-survivor) to find possible outliers. Data analysis was performed using the "centered" and "scaled" functions of the software. Q and T2 were used as statistical methods to detect possible outliers using the Influence plot, considering all PCs that explained a variance ≥5%. The confidence interval was settled at 95%. The discriminant variables were chosen by considering the value of the loadings (loading ≥±0.3).

Table S3. Clinical and biochemical parameters in survivors versus non-survivors at inclusion. Data are reported as percentage, mean ± standard deviation or median [interquartile range] as appropriate. P value: for the comparison between survivors vs non-survivors. WBC: white blood cells. mcL: million per microliter. U/L: units per liter. FEU: fibrinogen equivalent unit.

	Survivors (n=15)	Non-survivors (n=15)	p- value (α: 0.05)
Respiratory parameters at inclusion			
P/F ratio	151 ± 85	114 ±46	0.18
PaO2, (mmHg)	89 ±29	81± 25	0.46
PaCO2, (mmHg)	44± 12	51 ±19	0.21
spO2 ahb	96 [95-97]	94 [92-95]	0.02
PEEP (positive end-expiratory pressure therapy) cmH2O	10 ±2	11 ±2	0.17
Laboratory data at inclusion			
WBC (u x103/L)	6.3 [9.0-12.9]	7.1 [9.0-12.0]	0.85
Lymphocytes, (u x 103/L)	0.8 [1.0-1.2]	0.5 [0.7-0.8]	0.02
Monocytes. (u x 103/L)	0.9±0.4	0.5±0.4	0.03
Basophils (u x 103/L)	0.01 [0.03-0.05]	0.01 [0.02-0.04]	0.35
Red blood cells (u x 103/L)	3.4±0.6	3.8±0.6	0.10
Hematocrit (%)	31±4	34±5	0.04
Hemoglobin (g/dL)	10±1	11±2	0.05
Creatinine clearance (mg/dL)	0.6 [0.6-1.3]	0.90 [1.3-2.300]	0.03
Bilirubin (mg/dL)	0.5 [0.7-0.9]	0.4 [0.7-2.9]	0.61

Albumin (g/dL)	2.5 [2.6-2.8]	2.1 [2.2-2.5]	0.07
ALT (U/L)	26.5 [41.0-82.0]	28.7 [48.0-94.0]	0.88
LDH (U/L)	351±141	381±71	0.49
D-dimer (mg FEU/L)	1.4 [2.9-4.6]	1.8 [3.1-5.0]	0.84
C-reactive protein (mg/L)	3.3 [12.0-21.0]	12.3 [15.9-29.7]	0.11
Procalcitonin (ng/mL)	0.17 [0.25-0.67]	0.55 [0.75-3.47]	0.01
Tissue factor (ng/mL)	0.25 [0.38-0.52]	0.31 [0.68-1.04]	0.04
Thrombomodulin (ng/mL)	5.2 [7.8-16.3]	7.1 [12.9-20.6]	0.16
PAI-1	50.4 [66.4-103.3]	96.4 [146.7-185.5]	0.01
Lactic acid (mmol/L)	0.70 [0.90-1.20]	1.4 [1.7-2.0]	0.002
ADP-20 induced max platelet aggregation	10.4 [23.5-37.0]	7.9 [17.2-23.6]	0.41
Ca2+ (mg/dL)	8.3±0.6	8.6±0.4	0.11
K+ (mmol/L)	4.0±0.4	4.3±0.4	0.07
Biomarkers at inclusion			
IL-6 (pg/mL)	17.2 [33.9-1238]	25.2 [42.9-145.5]	0.84
IL-10 (pg/mL)	4.4 [6.0-9.1]	19.4 [36.0-70.2]	0.0002
sVCAM-1 (ng/mL)	788.7 [949.4-1114]	1412 [1585-2191]	0.002
TNF-α (pg/mL)	21.5 [40.2-71.4]	26.2 [40.9-71.2]	0.97

Table S4. Untargeted metabolomics results for GLCA-3S and GCDCA-3S stratified by patient (at T1, T2, T3)

	GLCA-3S			GCDCA-3S		
	T1	T2	T3	T1	T2	T3
S8	$3.94x10^{6}$	$1.59x10^{7}$	Not available	$4.00x10^{7}$	$2.00x10^{8}$	Not available
S9	$2.88x10^{6}$	Not available	$3.09x10^{6}$	$2.00x10^{7}$	Not available	$4.00x10^{7}$
S11	$4.26x10^{6}$	$5.25x10^{6}$	$4.59x10^{6}$	$4.00x10^{7}$	$8.00x10^{7}$	$8.00x10^{7}$
S14	$1.28x10^{8}$	$2.59Ex10^{7}$	Not available	$8.00x10^{8}$	$1.00x10^{7}$	
S15	$9.37x10^{7}$	$3.15x10^{7}$	$1.56x10^{6}$	$5.00x10^{9}$	$4.00 x10^{8}$	$3.00x10^{7}$
S22	$3.41x10^{7}$	$3.61 x10^{6}$	$5.64x10^{7}$	$3.00 x10^{9}$	$1.00 x10^{8}$	$8.00x10^{6}$
S24	Not available	$3.5x10^{7}$	$5.65x10^{6}$	Not available	$3.00 x10^{8}$	$2.00 x10^{8}$
S26	$4.42x10^{6}$	$2.28x10^{6}$	Not available	$1.00 x10^{9}$	$9.00x10^{7}$	Not available
S27	$1.15x10^{6}$	$3.71x10^{6}$	$1.58x10^{6}$	$4.00 x10^{7}$	$2.00 x10^{8}$	$8.00x10^{7}$
S29	Not available	$1.06 x10^{8}$	$3.64 x10^{8}$	Not available	$1.00 x10^{8}$	$8.00x10^{6}$

S31	Not available	Not available	$4.98x10^7$	Not available	Not available	$2.00x10^7$
S37	$7.91x10^5$	Not available	$8.90\ x10^5$	$2.00\ x10^7$	Not available	$1.00x10^7$
S48	$1.07x10^6$	Not available	Not available	$1.00\ x10^8$	Not available	Not available
S18	$5.19x10^7$	$3.36x10^7$	Not available	$3.00\ x10^8$	$5.00x10^6$	Not available
S21	Not available	$1.30x10^7$	$1.76x10^6$	Not available	$2.00x10^8$	$1.00x10^7$

Figure S2. Serum GCDCA-3S comparison between untargeted and targeted metabolomics for each patient

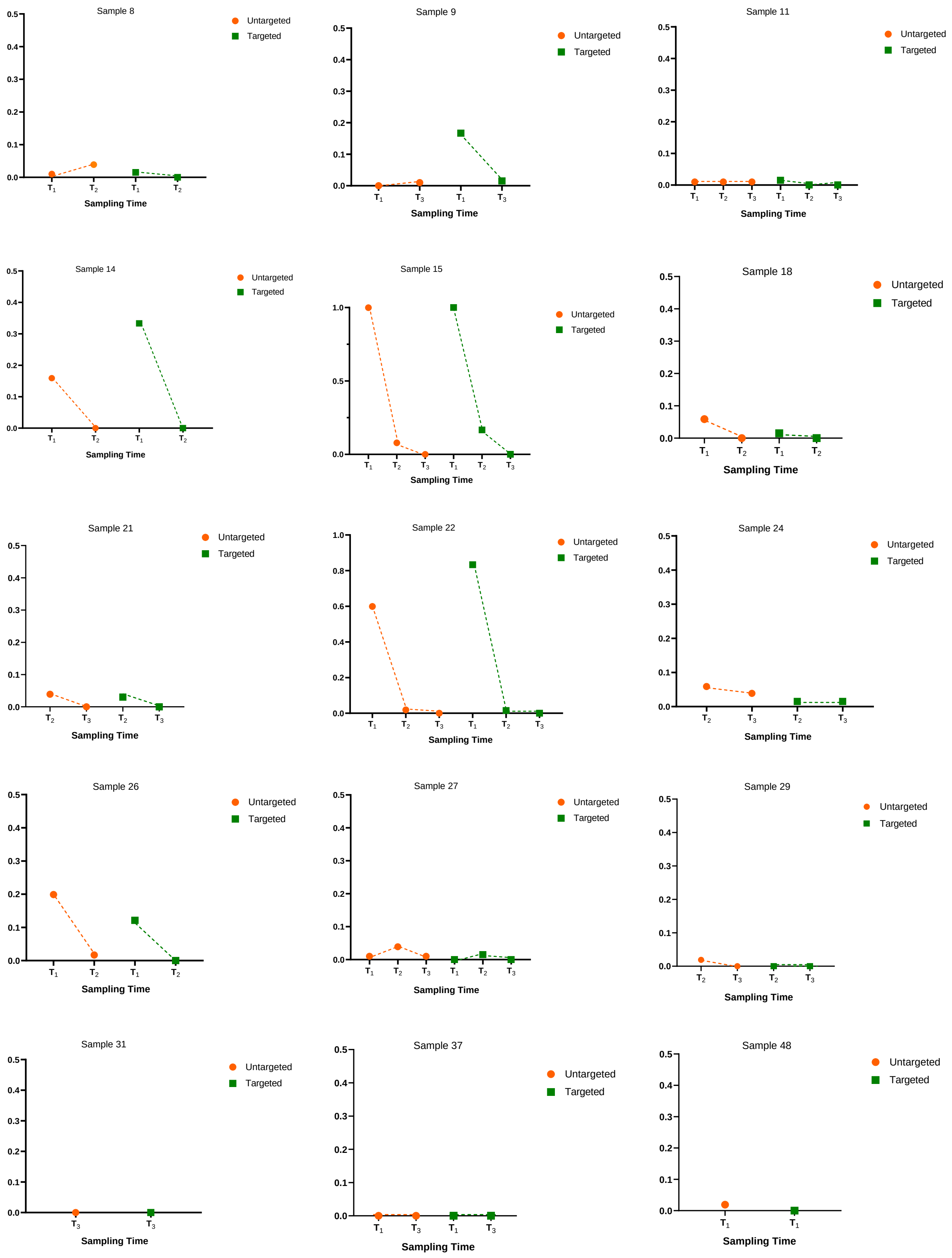

Figure S3. Serum GLCA-3S comparison between untargeted and targeted metabolomics for each patient

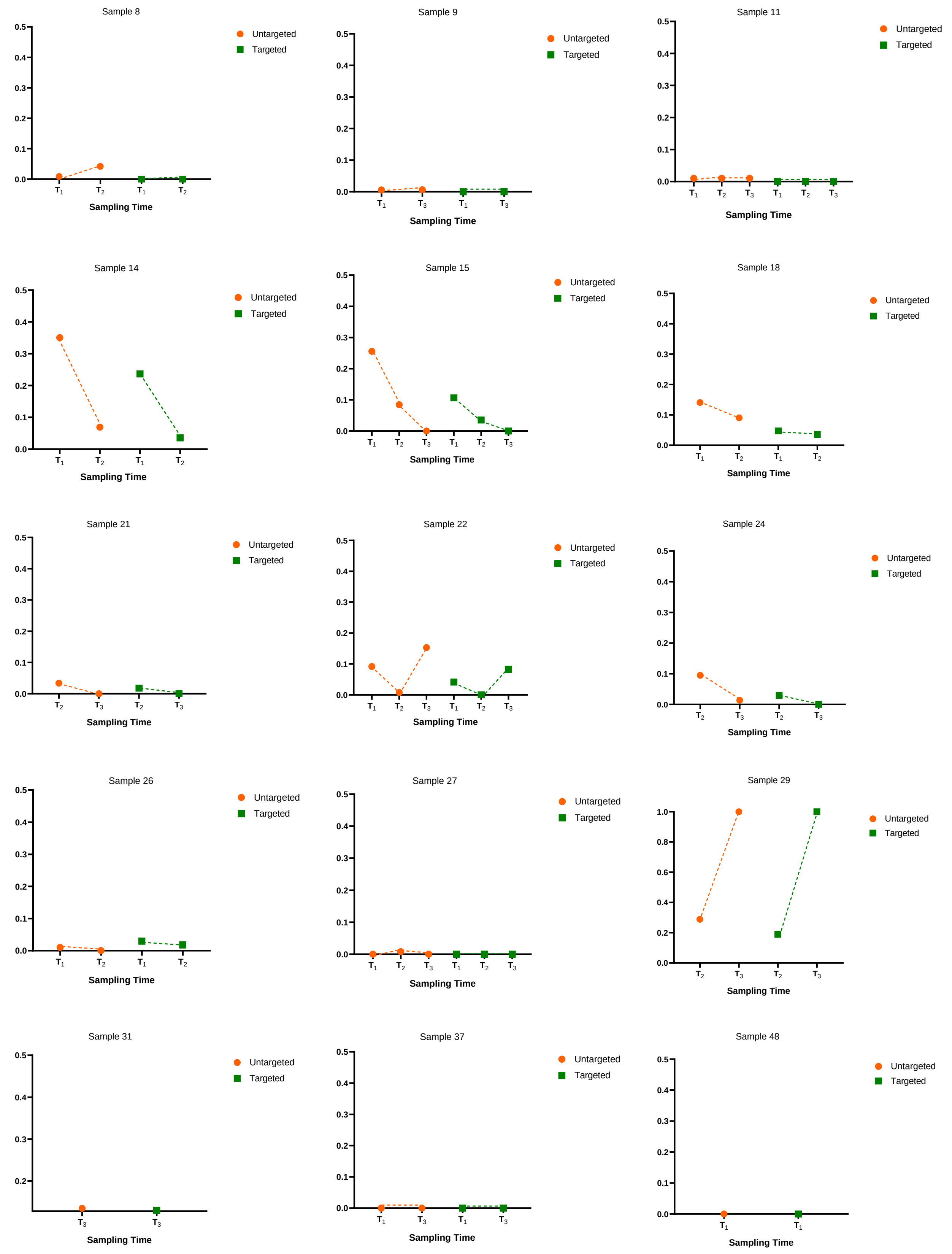

Table S5. Bile acid concentration in survivors and non-survivors patients (mean ± standard deviation). From the total database we exclude one non survivors who had colon cancer.

Bile acid	Survivors (n=15) (µM)	Non-survivors (n=14) (µM)
CA	0.2 ± 0.2	0.2± 0.3
GCA	1± 2	2 ±2
TCA	0.2± 0.5	0.1 ±0.2
CDCA	0.1 ±0.2	0.04 ±0.08
GCDCA	1 ±1	0.5 ±0.4
TCDCA	0.3± 0.4	0.1 ±0.1
DCA	0.04± 0.1	0.04 ±0.1
GDCA	0.04± 0.1	0.05 ±0.1
TDCA	0.01 ±0.05	0.03± 0.06
LCA	< LOD	< LOD
GLCA	< LOD	< LOD
TLCA	< LOD	< LOD
UDCA	0.03 ±0.09	0.02 ±0.05
GUDCA	0.07 ±0.1	0.05 ±0.07
TUDCA	< LOD	0.01 ±0.03
GCDCA-3S	0.1± 0.2	0.7 ±1
GLCA-3S	0.06± 0.1	0.7 ±0.7
LCA-3S	< LOD	< LOD

Figure S4. Box and wishes plot of serum GLCA-3S/(GLCA-3S+GCDCA) in survivors and non-survivors at three sampling time. Median (line), mean (+), and minimum and maximum values.

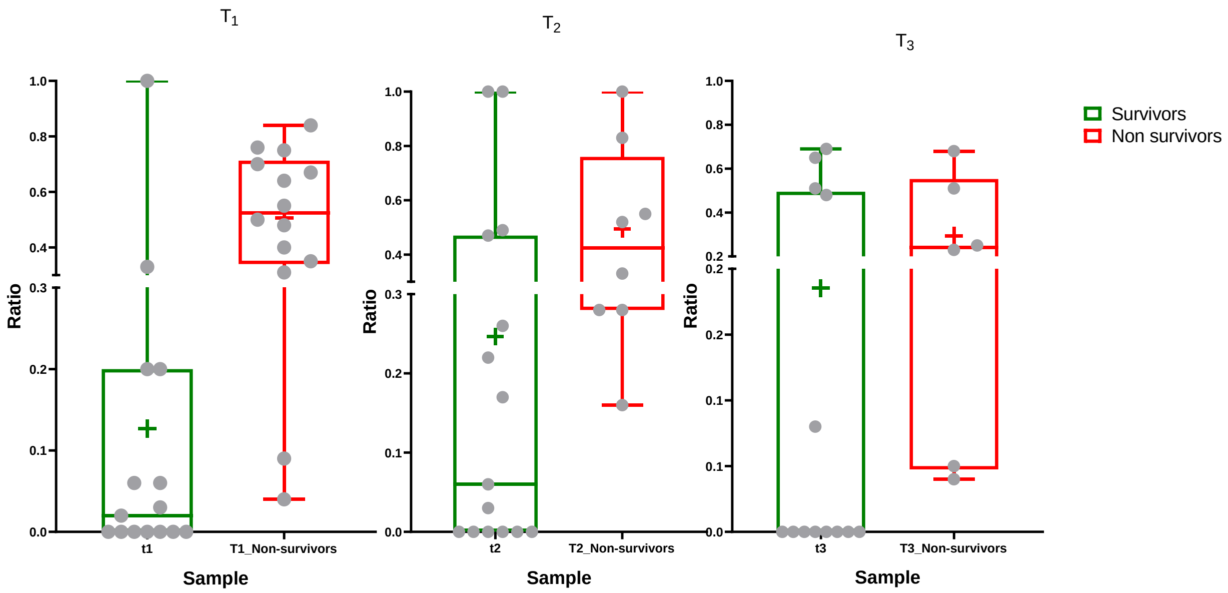

Figure S5. Box and wishes plot of Serum GCDCA-3S/(GCDCA-3S+GCDCA) in survivors and non-survivors at three sampling time. Median (line), mean (+) and minimum and maximum values.

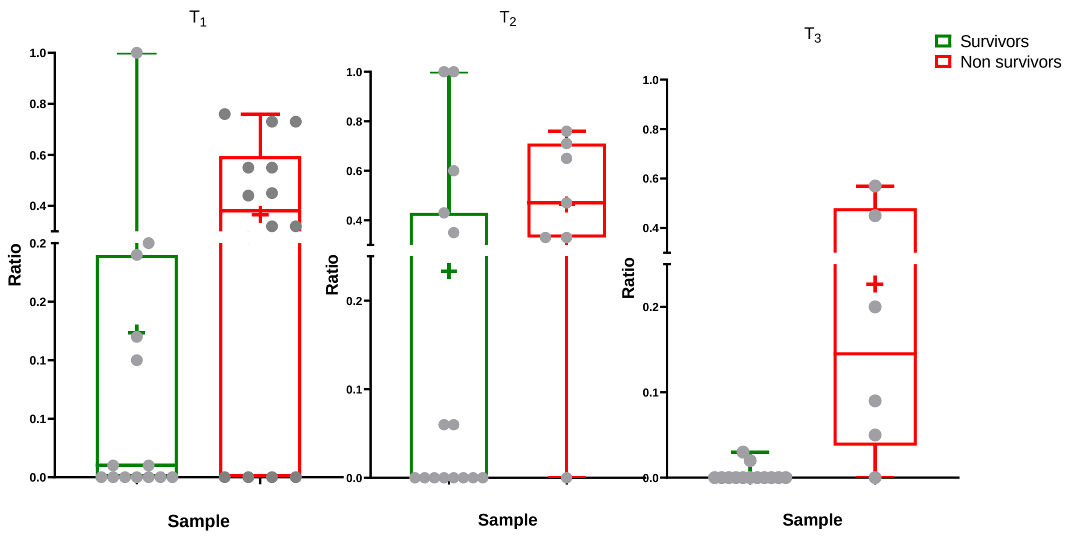

Figure S6. GCDCA, TCDCA, GCDCA-3S, and GLCA-3S concentrations in survivors and non-survivors at T1. Median (line), mean (+) and minimum and maximum values.

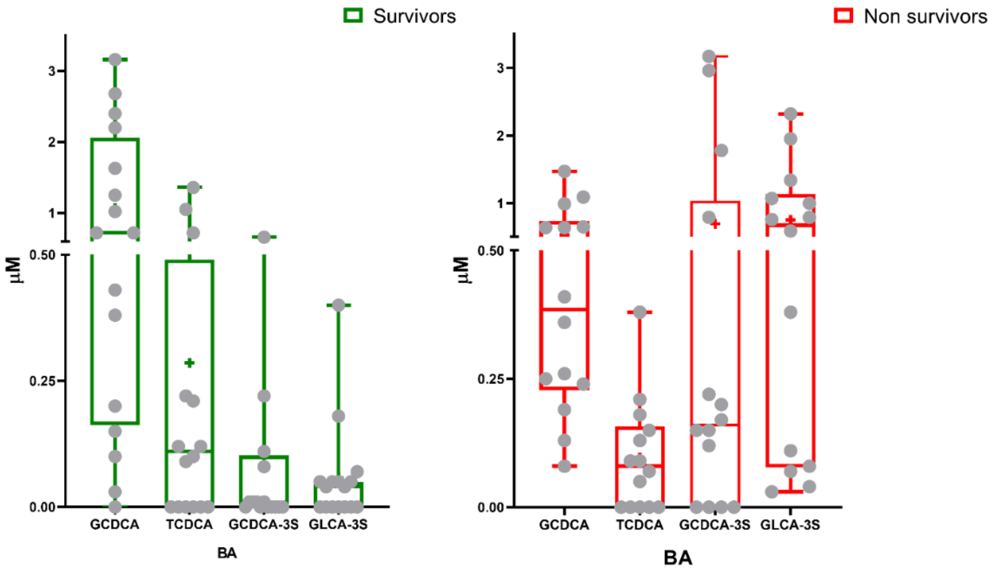

Table S6. Results of correlation matrix. Correlation coefficients and P-value

System	Clinical parameters	Sulfo-BA	Correlation coefficient (rS)	P-value
	Mortality	GLCA-3S	0.68	<0.001
		GLCA-3S/ (GCDCA+ GLCA-3S)	0.68	<0.001
		GCDCA-3S/ (GCDCA-3S + GCDCA)	0.41	0.02
Immune system	Lymphocytes	GLCA-3S	-0.53	0.003
		GLCA-3S/ (GCDCA+ GLCA-3S)	-0.66	<0.001
	Monocytes	GLCA-3S/ (GCDCA+ GLCA-3S)	-0.63	<0.001
		GLCA-3S	-0.44	0.02
	Basophils	GLCA-3S/ (GCDCA+ GLCA-3S)	-0.47	0.09
	Procalcitonin (PCT)	GLCA-3S	0.41	0.03

		GCDCA-3S	0.49	0.008
		GCDCA-3S/ (GCDCA-3S + GCDCA)	0.48	0.001
	Interleukin 10 (IL-10)	GLCA-3S	0.53	0.004
		GLCA-3S/ (GCDCA+ GLCA-3S)	0.48	0.009
	Tumor necrosis factor alpha (TNF-α)	GCDCA-3S/ (GCDCA+ GCDCA-3S)	0.48	0.007
Cardiovascular system	Red blood cells	GLCA-3S/ (GCDCA+ GLCA-3S)	0.49	0.006
	Hematocrit	GLCA-3S/ (GCDCA+ GLCA-3S)	0.51	0.004
	Hemoglobin	GLCA-3S/ (GCDCA+ GLCA-3S)	0.47	0.009
	Tissue factor	GLCA-3S/ (GCDCA+ GLCA-3S)	0.40	0.03
	Thrombomodulin	GCDCA-3S	0.41	0.02
	Plasminogen activator inhibitor-1 (PAI-1)	GLCA-3S	0.47	0.008
		GLCA-3S/ (GCDCA+ GLCA-3S)	0.53	0.002
	Soluble Vascular Cell Adhesion Molecule-1 (s-VCAM)	GLCA-3S/ (GCDCA+ GLCA-3S)	0.40	0.03
	ADP 20 -induced max platelet aggregation	GLCA-3S	-0.41	0.02
	Lactic acid	GLCA-3S	0.53	0.004
		GLCA-3S/ (GCDCA+ GLCA-3S)	0.59	0.001
Kidney	Potassium	GCDCA-3S	0.44	0.02
		GCDCA-3S/ (GCDCA-3S + GCDCA)	0.44	0.02
	Calcium	GLCA-3S/ (GCDCA+ GLCA-3S)	0.41	0.02
Liver	Bilirubine	GLCA-3S	0.61	0.001
		GCDCA-3S	0.71	<0.001
		GCDCA-3S/ (GCDCA-3S + GCDCA)	0.57	0.002
Lung	Positive end-expiratory pressure (PEEP)	GLCA-3S	0.44	0.03

Table S7. Logistic regression results for the association between BA-S concentrations and mortality.

Compound	Cutpoint	Sensitivity	Specitivity	Odd Ratio (95% CI)	p-value
GLCA-3S	0.26	85.71%	81.25%	26 (3.68-183.42)	0.001
GLCA-3S ratio with metabolic precursor	0.48	85.71%	87.50%	42 (5.1-345.1)	0.001
GCDCA-3S	0.40	71.43%	81.25%	10.8 (1.96-59.83)	0.006
GCDCA-3S ratio with metabolic precursor	0.51	71.43%	93.75%	37.5 (3.64-386.51)	0.002

Figure S7. Score plot (a) and loading plot (b) for PC2 vs PC3

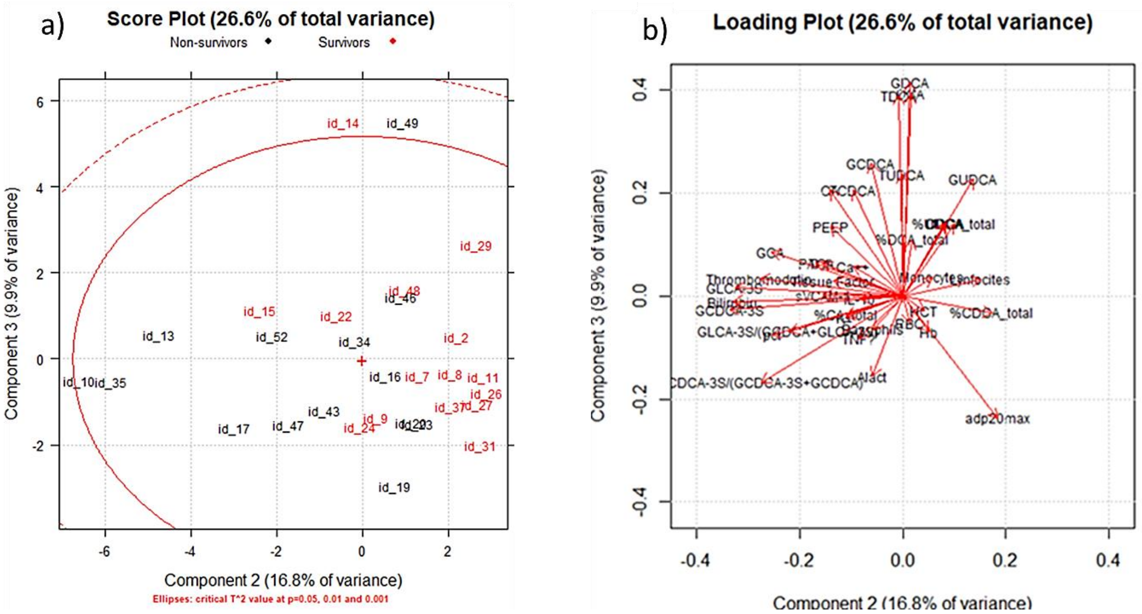

Figure S8. Influence Plot performed using 6 principal components.

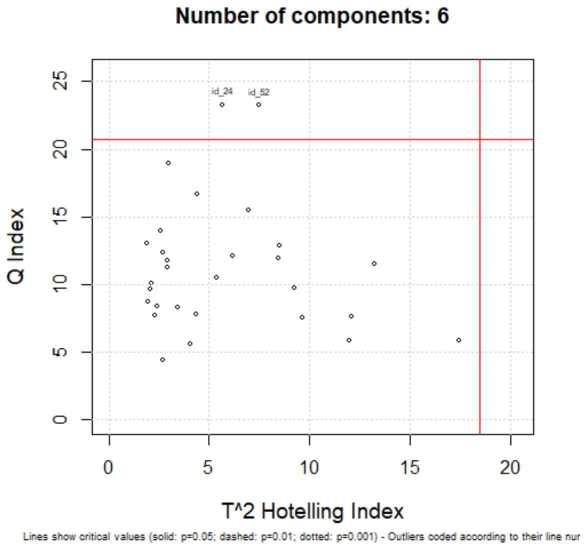

Figure S9. Box and wishes plot of serum total BA at T1 in survivors and non-survivors. Median (line), mean (+) and minimum and maximum values.

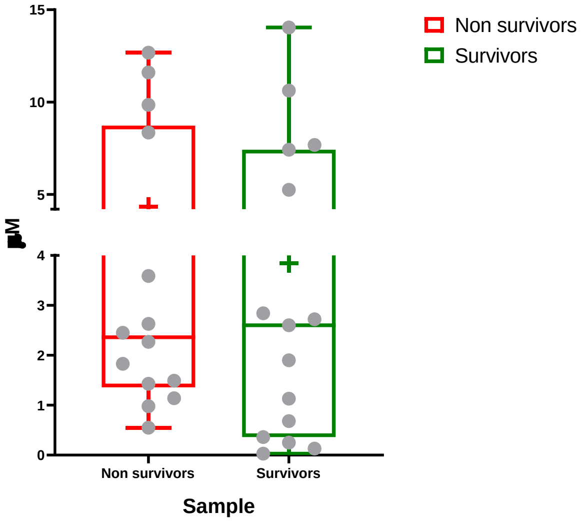

Supplement: Supplementary file 1 [file cells-13-01576-s001.zip › cells-3174028-supplementary.pdf]
